# A Case of Abortion in the Third Month

**Published:** 1889-04

**Authors:** Elbert Wing

**Affiliations:** Demonstrator of Pathology in the Chicago Medical College; 3266 Cottage Grove Avenue


### A CASE OF ABORTION IN THE THIRD MONTH.

BY ELBERT WING, A. M., M. D.

DEMONSTRATOR OF PATHOLOGY IN THE CHICAGO MEDICAL COLLEGE.

The recently published edition of Skeene's "Diseases of Women" contains a large number of reports of cases which are described in such a manner that their clinical history is clearly shown. The value of such reports to the recent graduate, and to the practitioner who for any reason is inexperienced, can scarcely be over-estimated. In the experience of the writer, his greatest difficulties and perplexities have arisen from an inability to find reports of cases similar to those which for any reason have taxed his knowledge, tact or therapeutic resources. And this, too, despite a preliminary hospital service, both as interne, and post-graduate student, as ample and extended as the current opinion in regard to medical education demands. In gynæclogical work, this demand for clinical histories is admirably met in the book already mentioned, but in obstetrics, neither the translation of Charpentier published by Wood & Co., nor Parvin's "Science and Art of Obstetrics," are of any assistance in this particular. This clinical report, therefore, is furnished for two reasons, viz: To show the favorable course of the temperature without the use of medicines, and in the hope that it may be of value to any practitioner, equally or even more inexperienced than the writer, who may chance to read it. And in the interest of brevity only pertinent facts are mentioned.

The patient is twenty years old, married, has one child eighteen months old, has had one prior miscarriage, and always during her menstrual life the catamenial periods have been very irregular.

In the present instance professional aid was first sought at 2:30 A. M., March 1st and the statement then made and the conditions found were as follows:

Menstruation had not appeared for "several months," (patient's statement), and with the intention of bringing it on the patient took a Turkish bath a few days before this date. During the afternoon of February 28th, uterine hemorrhage began and shortly became associated with uterine contractions. Medical assistance was evidently sought for the purpose of arresting the hemorrhage rather than the abortion.

Examination revealed the loss of considerable quantities of blood, a moderate uterine hemorrhage persisting, and the patient pallid but in good spirits. After removing the clots of blood from the vagina the placental membranes were felt protruding from the external os and lying partly in the vagina; the os and canal of the cervix would not admit the index finger. With the aid of a Sims' speculum the vagina was carefully dried with absorbent cotton, and a tampon placed which entirely filled and distended it. The pieces of cotton placed against and about the cervix were dipped in an aqueous solution of carbolic acid, two and one-half per centum, and squeezed partly dry before their introduction. A fœtus at the third month was found in the clots.

The patient was next seen at eleven o'clock, eight hours after the tampon was placed. There had been no escape of blood from the vagina, and only a few of the balls of cotton were stained, and these were odorless. A part of the placenta, connected with the rest of that organ, and about one inch long, lay in the vagina protruding from the external os. The index finger was easily passed through the internal os and the placenta found adherent about the left corner of the uterus. Under anæsthesia induced by Squibb's ether, the uterus was drawn down with volsella forceps fastened in the anterior cervical lip. Although the finger was easily introduced into the cavity of the uterus, it was very difficult to completely remove the placental fragments, and the conventional suggestion to use only gentle force could not be followed. A point of especial interest in this connection is the very marked superiority of Emmet's curette forceps over the finger or any of the various forms of dull curettes for removing the placental debris from the cavity of the body of the uterus. At the close of the curettement the intra-uterine douche was not employed because of the apparently complete removal of all placental tissue, and of the absence of hæmorrhage. By means of cotton wrapped upon an applicator, a mixture of equal parts of the tinctures of the chloride of iron, and of iodine, was freely applied to the cavity of the uterus. A copious vaginal douche of simple warm water was next given, and without either uterine or vaginal tampon the patient was put to bed with an ice-bag upon the hypogastrium. Careful directions were given to prepare and apply over the external genitalia and perinæum cloths dipped in a 1 to 1,000 solution of mercuric chloride. The nurse, who was untrained and not reliable, was also instructed to wash her hands with hot water, soap and a nail-brush both before and after changing the perineal cloths, and to use a 1 to 4,000 mercuric chloride solution in bathing the patient's perineal region and vulva.

The following copy of the clinical notes taken in the case will sufficiently explain its course from this stage:

March 1.—8 P. M. P. 112, T. 102.6°. Patient has had and has now only a very little pain either in the pelvic organs or elsewhere, and the amount of tenderness on palpation over the hypogastric and iliac regions is very slight. No tympanites; no vaginal discharge.

March 2.—2 P.M. P. 108, T. 103°. Conditions are substantially those of yesterday except a slight vaginal discharge which has no odor.

March 3.—Noon. P. 96, T. 100.3°. The nurse reports a slight odor about the soiled napkins. In other respects the conditions of yesterday are unchanged. Directions given for a vaginal douche twice daily of a 1 to 4,000 mercuric chloride solution.

March 4.—3 P. M. P. 96, T. 100.2°. Bowels moved twice since yesterday. Conditions otherwise unchanged.

March 5.—3 P. M. P. 84, T. 99°. Bowels moved once. Vaginal discharge has no odor. Tenderness on palpation over abdomen scarcely more than normal. The appearance of the patient is that of health and comfort, and she says that she feels perfectly well. The use of ice over the hypogastrium stopped.

March 7.—2 P. M. P. 84, T. 98.6°. There is still a very slight odorless vaginal discharge. The patient declares herself free from pain and discomfort and wants to get up.

With the customary directions in regard to caution in resuming her usual occupation, the patient is discharged.

The temperatures given were taken in the mouth with a Hicks' thermometer which has been twice corrected and they are reliable.

3266 COTTAGE GROVE AVENUE.

